# Supplementary figures and images for: Assembly and comparative analysis of complete mitochondrial genome sequence of an economic plant Salix suchowensis
Source: PeerJ. 2017 Mar 29;5:e3148. doi: 10.7717/peerj.3148 (PMC5374973; doi:10.7717/peerj.3148)

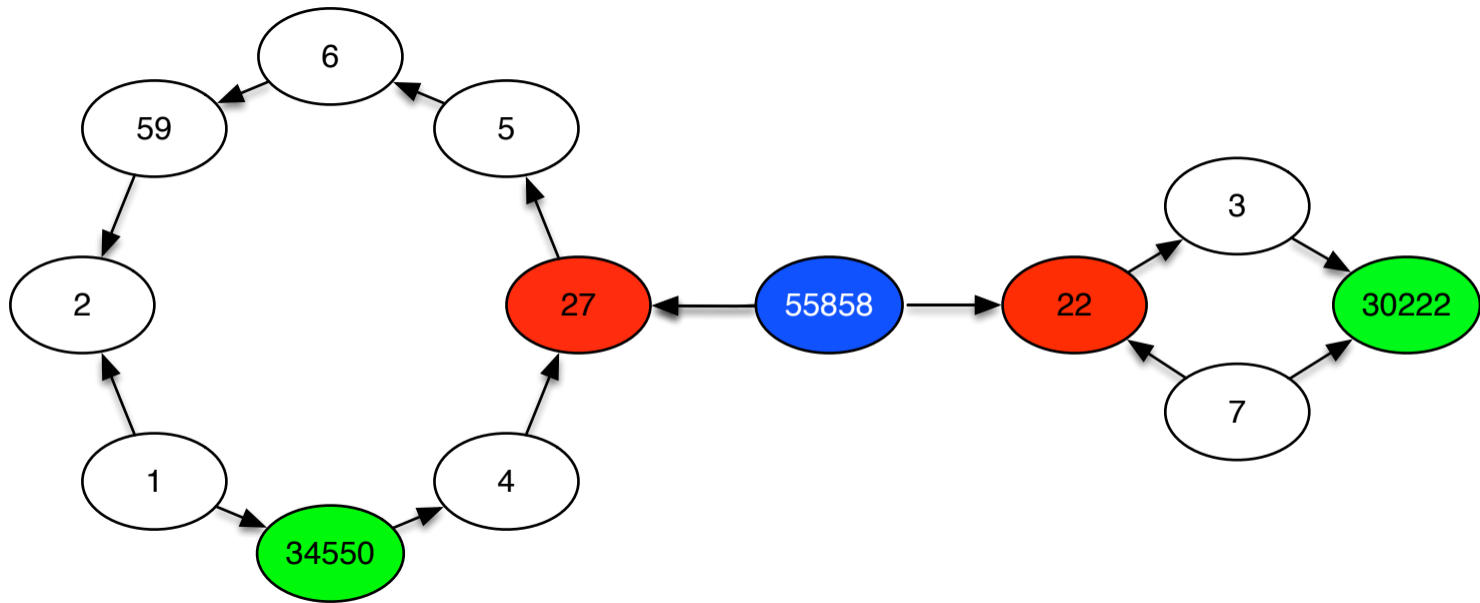

Supplement: Figure S1 — The de novo assembled graph of S. suchowensis mt genome. The ellipses stand for assembled contigs and the numbers in the ellipses show the contig names. The arrows between two contigs stand for the assembled orientation. The ellipses in green show contigs derived from S. suchowensis cp genome, the red ellipses show repeat contigs, and the blue one shows the contigs are both cp-derived and repeat contigs. The figure was generated with OmniGraffle. [file peerj-05-3148-s001.pdf]
