# Supplementary material for: Assembly and comparative analysis of complete mitochondrial genome sequence of an economic plant Salix suchowensis
Source: PeerJ. 2017 Mar 29;5:e3148. doi: 10.7717/peerj.3148 (PMC5374973; doi:10.7717/peerj.3148)
Supplement: Figure S2 — The detailed location of mt genome, GC content, CDs, tRNA, and other useful information are illustrated in this GBrowse. [file peerj-05-3148-s002.pdf]

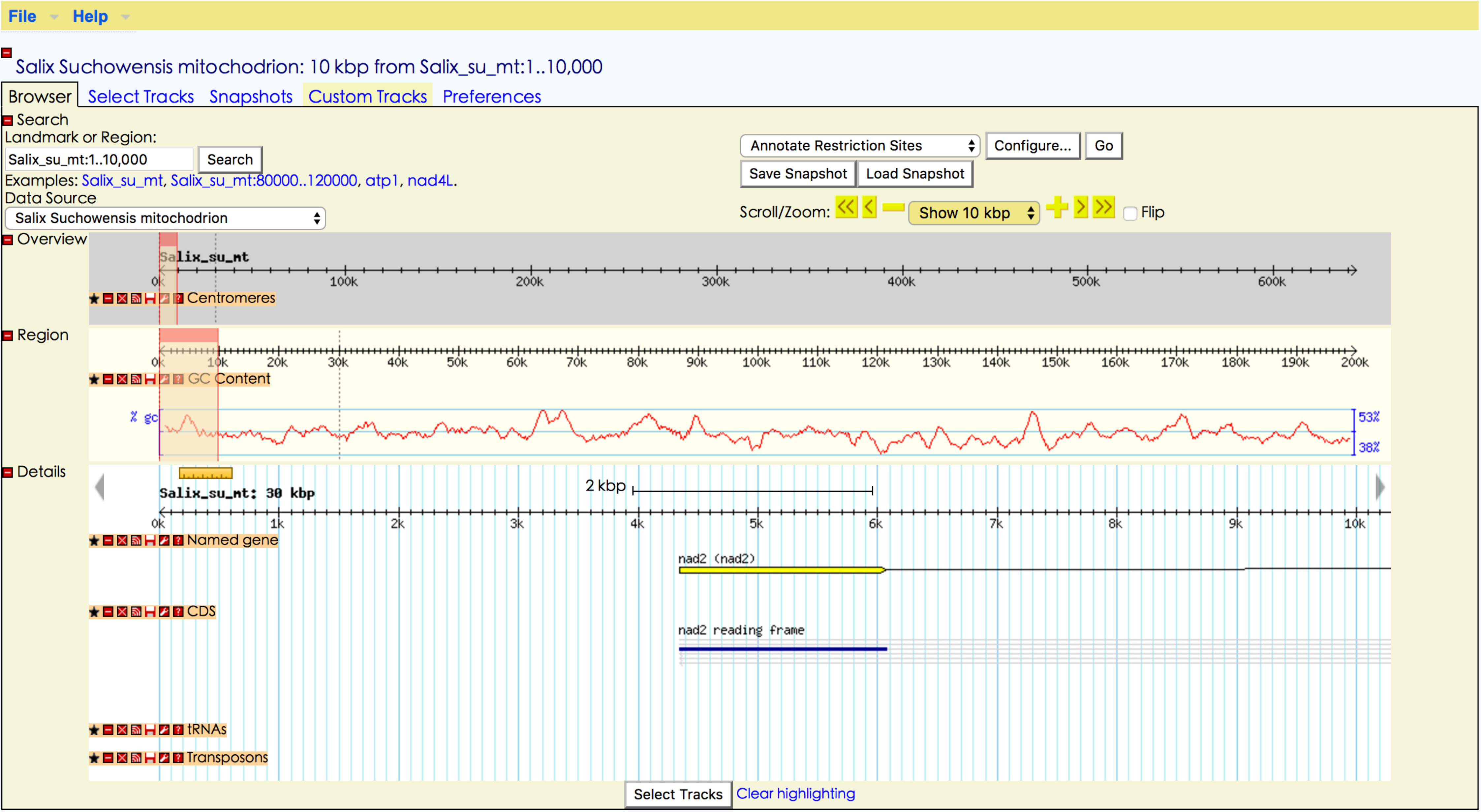
File
Help
Salix Suchowensis mitochodrion: 10 kbp from Salix_su_mt:1..10,000
Browser
Select Tracks
Snapshots
Custom Tracks
Preferences
Search
Landmark or Region:
Salix_su_mt:1..10,000
Search
Examples: Salix_su_mt, Salix_su_mt:80000..120000, atp1, nad4L.
Data Source
Salix Suchowensis mitochodrion
Annotate Restriction Sites
Configure...
Go
Save Snapshot
Load Snapshot
Scroll/Zoom:
Show 10 kbp
Flip
Overview
Salix_su_mt
0k 100k 200k 300k 400k 500k 600k
Centromeres
Region
0k 10k 20k 30k 40k 50k 60k 70k 80k 90k 100k 110k 120k 130k 140k 150k 160k 170k 180k 190k 200k
GC Content
% gc
53%
38%
Details
Salix_su_mt: 30 kbp
2 kbp
0k 1k 2k 3k 4k 5k 6k 7k 8k 9k 10k
Named gene
nad2 (nad2)
CDS
nad2 reading frame
tRNAs
Transposons
Select Tracks
Clear highlighting
