# Supplementary material for: Assembly and comparative analysis of complete mitochondrial genome sequence of an economic plant Salix suchowensis
Source: PeerJ. 2017 Mar 29;5:e3148. doi: 10.7717/peerj.3148 (PMC5374973; doi:10.7717/peerj.3148)
Supplement: Table S1 [file peerj-05-3148-s003.docx]

**Table S1. Statistics of the genome assembly in *S. suchowenisis* mt genome**

| Statistical list | **Number** |
| --- | --- |
| Number of raw reads | 1,240,387 |
| Total length of raw reads (bp) | 702,204,081 |
| Average raw read length (bp) | 567 |
| Longest raw read (bp) | 1,201 |
| Number of assembled contigs | 235,005 |
| Longest contig (bp) | 349,758 |
| Number of read depth (50x-100x) | 296 |
| Total number (>=10 kb) | 13 |
| Total number (>=1 kb) | 21,662 |
