# Supplementary material for: Assembly and comparative analysis of complete mitochondrial genome sequence of an economic plant Salix suchowensis
Source: PeerJ. 2017 Mar 29;5:e3148. doi: 10.7717/peerj.3148 (PMC5374973; doi:10.7717/peerj.3148)
Supplement: Table S2 [file peerj-05-3148-s004.docx]

**Table S2.** **Assembled contigs of *S. suchowensis* mt genome.**

| **No.** | **Name** | **Coverage** | **Number of reads** | **Length (bp)** |
| --- | --- | --- | --- | --- |
| 1 | Contig00001 | 22.3 | 17,353 | 349,730 |
| 2 | Contig00002 | 21 | 4,053 | 85,613 |
| 3 | Contig00003 | 22.8 | 2,366 | 46,498 |
| 4 | Contig00004 | 22.2 | 1,814 | 37,000 |
| 5 | Contig00005 | 20.3 | 1,507 | 33,187 |
| 6 | Contig00006 | 20.2 | 1,262 | 28,005 |
| 7 | Contig00007 | 21.6 | 1,247 | 24,942 |
| 8 | Contig00022 | 43.5 | 787 | 7,666 |
| 9 | Contig00027 | 42 | 765 | 7,518 |
| 10 | Contig00059 | 16.2 | 208 | 6,458 |
| 11 | Contig30222 | 634.4 | 1,969 | 912 |
| 12 | Contig34550 | 751.2 | 2,046 | 814 |
| 13 | Contig55858 | 1482.7 | 2,695 | 397 |
|  | Total/Average | 25.2^*^ | 2,929 | 628,740 |

^*^The average coverage of 10 aligned contigs without three cp-derived contigs: Contig30222, Contig34550 and Contig55858.
