# Supplementary material for: Assembly and comparative analysis of complete mitochondrial genome sequence of an economic plant Salix suchowensis
Source: PeerJ. 2017 Mar 29;5:e3148. doi: 10.7717/peerj.3148 (PMC5374973; doi:10.7717/peerj.3148)
Supplement: Table S3 [file peerj-05-3148-s005.docx]

**Table S3. The abbreviations and NCBI accession numbers of all used plant mt genomes.**

| **Species** | **Abbreviations** | **Accession Numbers** |
| --- | --- | --- |
| *Ajuga reptans* | *A. reptans* | NC_023103 |
| *Asclepias syriaca* | *A. syriaca* | NC_022796 |
| *Arabidopsis thaliana* | *A. thaliana* | NC_001284 |
| *Bartramia pomiformis* | *B. pomiformis* | NC_024519 |
| *Batis maritima* | *B. maritima* | NC_024429 |
| *Beta vulgaris* | *B. vulgaris* | NC_015099 |
| *Brassica napus* | *B. napus* | NC_008285 |
| *Butomus umbellatus* | *B. umbellatus* | NC_021399 |
| *Buxbaumia aphylla* | *B. aphylla* | NC_024518 |
| *Capsicum annuum* | *C. annuum* | NC_024624 |
| *Carica papaya* | *C. papaya* | NC_012116 |
| *Citrullus lanatus* | *C. lanatus* | NC_014043 |
| *Cucumis sativus* | *C. sativus* | NC_016005 |
| *Cucurbita pepo* | *C. pepo* | NC_014050 |
| *Cycas taitungensis* | *C. taitungensis* | NC_010303 |
| *Daucus carota* | *D. carota* | NC_017855 |
| *Ginkgo biloba* | *G. biloba* | NC_027976 |
| *Glycine max* | *G. max* | NC_020455 |
| *Gossypium hirsutum* | *G. hirsutum* | NC_027406 |
| *Gossypium raimondii* | *G. raimondii* | NC_027407 |
| *Lotus japonicas* | *L. japonicas* | NC_016743 |
| *Malus domestica* | *M. domestica* | NC_018554 |
| *Marchantia polymorpha* | *M. polymorpha* | NC_001660 |
| *Millettia pinnata* | *M. pinnata* | NC_016742 |
| *Medicago truncatula* | *M. truncatula* | NC_029641 |
| *Nicotiana tabacum* | *N. tabacum* | NC_006581 |
| *Oryza sativa* | *O. sativa* | NC_007886 |
| *Phoenix dactylifera* | *P. dactylifera* | NC_016740 |
| *Populus tremula* | *P. tremula* | NC_028096 |
| *Raphanus sativus* | *R. sativus* | NC_018551 |
| *Rhazya stricta* | *R. stricta* | NC_024293 |
| *Salix suchowensis* | *S. suchowensis* | NC_029317 |
| *Salvia miltiorrhiza* | *S. miltiorrhiza* | NC_023209 |
| *Sorghum bicolor* | *S. bicolor* | NC_008360 |
| *Sphagnum palustre* | *S. palustre* | NC_024521 |
| *Treubia lacunosa* | *T. lacunosa* | NC_016122 |
| *Tripsacum dactyloides* | *T. dactyloides* | NC_008362 |
| *Triticum aestivum* | *T. aestivum* | NC_007579 |
| *Vigna angularis* | *V. angularis* | NC_021092 |
| *Vitis vinifera* | *V. vinifera* | NC_012119 |
| *Zea mays* | *Z. mays* | NC_008332 |
