# Supplementary material for: Assembly and comparative analysis of complete mitochondrial genome sequence of an economic plant Salix suchowensis
Source: PeerJ. 2017 Mar 29;5:e3148. doi: 10.7717/peerj.3148 (PMC5374973; doi:10.7717/peerj.3148)
Supplement: Table S4 [file peerj-05-3148-s006.docx]

**Table S4. Syntenic blocks (>1000 bp) between *S. purpurea* and *S. suchowensis* mt genomes**

| **Blocks** | **Length (bp)** | **Identity (％)** | **Location** | |
| --- | --- | --- | --- | --- |
|  |  |  | ***S. purpurea*** | ***S. suchowensis*** |
| **1** | 44,758 | 99.69 | 1-44,708 | 55,515-100,208 |
| **2** | 14,959 | 99.67 | 47,749-62,679 | 177,774-192,715 |
| **3** | 3,830 | 99.77 | 62,676-66,497 | 19,680-15,851 |
| **4** | 6,876 | 99.62 | 66,498-73,357 | 348,262-355,131 |
| **5** | 1,543 | 100 | 73,353-74,895 | 31,535-33,077 |
| **6** | 12,233 | 99.86 | 78,513-90,734 | 33,075-45,306 |
| **7** | 4,233 | 99.29 | 90,738-94,954 | 177,788-173,569 |
| **8** | 7,487 | 99.41 | 94,949-102,416 | 173,512-166,047 |
| **9** | 33,990 | 99.72 | 10,2454-136,386 | 558,365-592,324 |
| **10** | 9,835 | 99.53 | 136,385-146,194 | 101,287-111,103 |
| **11** | 34,285 | 99.59 | 146,185-180,392 | 131,821-166,055 |
| **12** | 1,854 | 99.62 | 180,935-182,788 | 556,478-558,326 |
| **13** | 37,205 | 99.84 | 182,782-219,948 | 519,301-556,493 |
| **14** | 51,449 | 99.65 | 204,363-255,722 | 644,437-593,026 |
| **15** | 6,580 | 99.09 | 256,687-263,238 | 286,903-280,350 |
| **16** | 1,100 | 100 | 263,704-264,803 | 277,704-276,605 |
| **17** | 80,267 | 99.72 | 264,804-344,973 | 275,254-195,090 |
| **18** | 11,660 | 99.5 | 344,834-356,453 | 11,646-1 |
| **19** | 7,399 | 99.68 | 357,470-364,850 | 327,351-319,958 |
| **20** | 27,769 | 99.63 | 368,177-395,912 | 287,619-315,326 |
| **21** | 10,981 | 99.33 | 395,840-406,772 | 518,173-507,209 |
| **22** | 1,002 | 99.8 | 406,781-407,782 | 355,107-356,104 |
| **23** | 3,508 | 99.6 | 407,779-411,280 | 30,917-27,411 |
| **24** | 143,567 | 99.67 | 411,082-554,388 | 358,370-501,773 |
| **25** | 2,734 | 95.32 | 568,211-570,912 | 501,773-504,437 |
| **26** | 21,371 | 99.56 | 572,506-593,852 | 348,659-327,343 |
| **27** | 1,086 | 100 | 595,161-596,246 | 101,293-100,208 |
